# Integrated Metabolomic and Bioenergetic Profiling Reveals Axl as a Key Regulator of Macrophage Homeostasis

**DOI:** 10.1002/eji.70293

**Published:** 2026-10-08

**Authors:** Jesuino R M Ferreira, Thais S Rigoni, Monique S Leandro, Kamila Guimarães‐Pinto, Letícia R Ramos, André L A Costa, Clara F Carvalho, Caroline F Martiniuc, Isadora A Oliveira, Carolina Maia, Júlia Moratelli, Jefferson A Leite, Verônica S Pinto, Adriane Todeschini, Antônio Galina, Alessandra A Filardy

**Affiliations:** ^1^ Laboratório de Imunologia Celular Instituto de Microbiologia Paulo de Góes Universidade Federal do Rio de Janeiro Rio de Janeiro Brazil; ^2^ Plataforma Bi‐Institucional de Pesquisa em Medicina Translacional FIOCRUZ SP/Faculdade de medicina de Ribeirão Preto Universidade de São Paulo São Paulo Brazil; ^3^ Laboratório de Bioenergética e Fisiologia Mitocondrial, Instituto de Bioquímica Médica Leopoldo de Meis Universidade Federal do Rio de Janeiro Rio de Janeiro Brazil; ^4^ Laboratório de Biotecnologia e Ecologia Microbiana, Instituto de Microbiologia Paulo de Góes Universidade Federal do Rio de Janeiro Rio de Janeiro Brazil; ^5^ Centro de Espectrometria de Massas de Biomoléculas (CEMBIO) Centro de Ciências da Saúde, Universidade Federal do Rio de Janeiro Rio de Janeiro Brazil; ^6^ Laboratório de Biologia de Linfócitos T, Instituto de Microbiologia Paulo de Góes Universidade Federal do Rio de Janeiro Rio de Janeiro Brazil; ^7^ Laboratório de Bioquímica do Exercício e Motores Moleculares Escola de Educação Física e Desportos (EEFD), Universidade Federal do Rio de Janeiro Rio de Janeiro Brazil; ^8^ Laboratório de Glicobiologia, Instituto de Biofísica Carlos Chagas Filho Universidade Federal do Rio de Janeiro Rio de Janeiro Brazil; ^9^ Instituto Nacional de Ciência e Tecnologia de Mucosa e Pele Universidade Federal de Minas Gerais Belo Horizonte Brazil

**Keywords:** Axl receptor, immunometabolism, oxidative stress, peritoneal macrophages

## Abstract

The TAM receptor Axl is essential for efferocytosis and inflammatory regulation, but its role in macrophage immunometabolic homeostasis remains unclear. Here, we combined phenotypic, functional, metabolomic, and bioenergetic analyses to define how Axl deficiency alters peritoneal macrophages (PMs) under steady‐state conditions. Axl^−^^/^^−^ PMs exhibited reduced cell numbers, downregulation of resident macrophage markers, and increased Ly6C expression, indicating a shift toward a less mature, monocyte‐like phenotype. Functionally, these cells showed impaired inflammatory activation, with reduced nitrite release, diminished LPS‐induced ROS production, and lower mitochondrial ROS. Metabolomic profiling revealed broad disruption of central carbon metabolism, including reduced glycolytic intermediates, depletion of tricarboxylic acid (TCA) cycle metabolites, and decreased NAD^+^/NADH and NADP^+^/NADPH pools. Despite reduced glycolytic flux, preserved pyruvate levels suggested compensatory carbon utilization. Bioenergetic assessment demonstrated pronounced mitochondrial dysfunction, characterized by reduced maximal respiration, elevated proton leak, and limited oxidative phosphorylation flexibility. Redox balance was also impaired, with decreased GSH, GSSG, and dehydroascorbic acid and increased ophthalmic acid, consistent with elevated oxidative stress. Systemically, Axl^−^^/^^−^ mice displayed hepatic redox imbalance and biochemical signs of liver dysfunction. Collectively, these findings identify Axl as a central regulator of macrophage identity, mitochondrial performance, and redox resilience, required for maintaining local and systemic homeostasis.

## Graphical abstract

Axl deficiency disrupts peritoneal macrophage identity and function, impairing glycolysis, TCA metabolism, and mitochondrial respiration while weakening antioxidant defenses. This coordinated metabolic and redox collapse extends systemically, highlighting Axl as a key regulator of immunometabolic homeostasis.

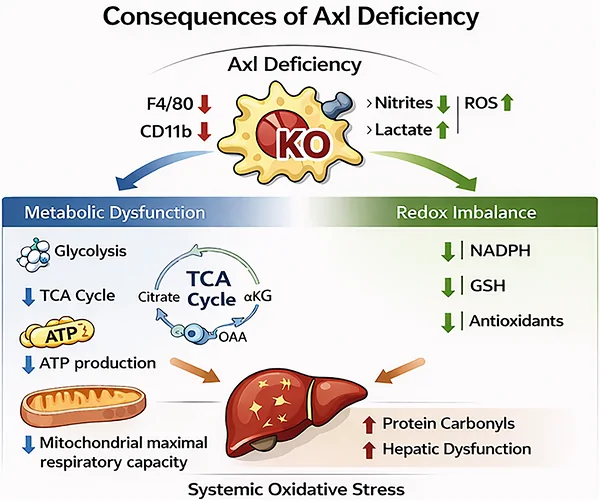

**Abbreviations**
ATP: adenosine triphosphate
DHE: dihydroethidium
FAD: flavin adenine dinucleotide
FADH_2_
: reduced flavin adenine dinucleotide
FAO: fatty acid oxidation
FCCP: carbonyl cyanide‐4‐(trifluoromethoxy) phenylhydrazone
GSH: reduced glutathione
GSSG: oxidized glutathione
LDH: lactate dehydrogenase
LPS: lipopolysaccharide
MFI: mean fluorescence intensity
NAD^+^
: nicotinamide adenine dinucleotide (oxidized form)
NADH: nicotinamide adenine dinucleotide (reduced form)
NADP^+^
: nicotinamide adenine dinucleotide phosphate (oxidized form)
NADPH: nicotinamide adenine dinucleotide phosphate (reduced form)
OAA: oxaloacetate
OXPHOS: oxidative phosphorylation
PBS: phosphate‐buffered saline
PCA: principal component analysis
PF: peritoneal fluid
PMs: peritoneal macrophages
ROS: reactive oxygen species
TBARS: thiobarbituric acid reactive substances
TCA: tricarboxylic acid
WT: wild type
αKG: α‐ketoglutarate
γ‐GT: γ‐glutamyl transferase

## 1 Introduction

Tissue‐resident macrophages are key regulators of immune surveillance and tissue homeostasis, relying on tightly coordinated metabolic programs to sustain their specialized functions. In the peritoneal cavity, large peritoneal macrophages (LPMs) and small peritoneal macrophages (SPMs) coexist in a dynamic equilibrium, with LPMs constituting the dominant long‐lived population under steady‐state conditions [1, 2]. Although ontogeny and functional heterogeneity of these subsets are well characterized, the molecular mechanisms that maintain their metabolic fitness and redox balance remain poorly defined.

Redox homeostasis is central to macrophage biology. Reactive oxygen species (ROS) function not only as antimicrobial effectors but also as signaling intermediates that modulate activation, polarization, and survival. Controlled ROS production, particularly hydrogen peroxide (H_2_O_2_), acts as a second messenger integrating transcriptional and metabolic responses to environmental cues [3, 4]. Mitochondria‐ and NADPH oxidase‐derived ROS influence macrophage polarization, with moderate ROS promoting pro‐inflammatory programs while antioxidant systems restrain oxidative injury during resolution phases [3, 4]. Excessive oxidative stress, however, impairs macrophage viability, disrupts phagocytic capacity, and limits self‐renewal of tissue‐resident macrophages by inducing metabolic collapse, senescence‐like states, or damage‐associated transcriptional responses [5, 6, 7, 8, 9]. Thus, maintaining a balanced redox environment is essential for sustaining macrophage effector functions and long‐term tissue residency.

Axl, a receptor tyrosine kinase of the TAM (Tyro3, Axl, MerTK) family, is well known for its roles in apoptotic cell clearance and resolution of inflammation [10, 11, 12]. Beyond its immunoregulatory functions, Axl contributes to cellular proliferation, survival, and migration, and its dysregulation is widely implicated in tumor progression [13, 14, 15]. However, the contribution of Axl to the metabolic and redox homeostasis of tissue‐resident macrophages has remained largely unexplored.

Macrophage metabolic programs are tightly coupled to redox control and fundamental functions such as phagocytosis, inflammatory regulation, and tissue adaptation. Under homeostatic conditions, resident macrophages are generally thought to rely on oxidative phosphorylation (OXPHOS) and fatty acid oxidation (FAO), metabolic pathways that support long‐term survival while limiting excessive ROS accumulation [16, 17, 18]. However, distinct tissue‐resident macrophage populations exhibit considerable metabolic heterogeneity, reflecting adaptations to their specific tissue microenvironments and functional demands [19]. Perturbations in mitochondrial activity or antioxidant systems disrupt this metabolic‐redox balance and trigger dysfunctional inflammatory states [20]. Although Axl is classically linked to efferocytosis and anti‐inflammatory signaling, emerging evidence indicates that TAM receptors can interface with cellular metabolic pathways. In macrophages, Axl limits ROS accumulation during apoptotic cell clearance [11], while studies in cancer cells, endothelial cells, and vascular smooth muscle cells reveal that Axl signaling can modulate glycolytic flux, mitochondrial activity, and oxidative stress responses [21, 22, 23, 24]. These findings raise the possibility that Axl may also govern metabolic fitness and redox regulation in tissue‐resident macrophages.

The growing recognition that metabolic reprogramming drives immune dysfunction highlights the need to elucidate pathways that maintain immune homeostasis [25]. Given the pronounced immunometabolic plasticity of peritoneal macrophages (PMs) [26, 27], defining the regulatory role of Axl in this tissue‐specific context may reveal fundamental mechanisms of local and systemic immune control [28]. Moreover, because metabolic and redox disruptions within macrophages can propagate to distal organs, understanding Axl function may illuminate previously unappreciated links between local immune alterations and whole‐body metabolic physiology.

Here, we demonstrate that Axl is a critical regulator of PM subset composition, metabolic flexibility, and antioxidant capacity. Axl deficiency reduced the PM pool and led to marked alterations in basal and stress‐induced metabolic profiles. Axl^−/−^ PMs exhibited increased glycolytic activity, diminished nitric oxide production, impaired adaptation to mitochondrial inhibition, and selective disruptions in glycolytic and tricarboxylic acid (TCA) cycle intermediates. Antioxidant defenses were significantly compromised, and metabolic and redox imbalances extended beyond the macrophage compartment, manifesting as oxidative stress and metabolic dysfunction in hepatic tissue. Together, these results identify Axl as a key coordinator of immunometabolic homeostasis, linking macrophage regulation to organism‐level metabolic integrity. These findings may hold important implications for the systemic effects of Axl‐targeted therapies currently used in oncology.

## 2 Results

### 2.1 Axl Deficiency Alters the Composition and Phenotype of Peritoneal Macrophages Under Homeostatic Conditions

To investigate PMs under homeostatic conditions, peritoneal fluid (PF) cells were analyzed by flow cytometry, and CD19^+^ B‐1 cells were excluded from the gating strategy (Figure 1A). PMs were identified based on CD11b and F4/80 expression [25], and full‐body (global) Axl^−/−^ mice exhibited a significant reduction in PMs compared with WT controls (Figure 1B,C). Next, based on MHCII and CD206 expression, PM subpopulations were analyzed, revealing a significant decrease in both the transitional CD206^+^MHCII^+^ PMs and the MHCII^+^ PM subset in Axl^−/−^ mice relative to WT animals (Figure 1D,F,G), which shows that the PM pool is partially dependent on Axl signaling that may be either cell‐intrinsic within PMs or indirect, mediated through other Axl‐expressing cell types within the peritoneal environment that can influence PM composition and phenotype. Given that MHCII^+^ macrophages are commonly associated with a more inflammatory, proinflammatory‐like profile, whereas CD206^+^ cells display features of alternatively activated, anti‐inflammatory‐like macrophages, these data suggest that Axl contributes to the homeostatic balance among phenotypically distinct PM subsets [29]. Further phenotypic characterization demonstrated a significant reduction in the mean fluorescence intensity (MFI) of the resident macrophage‐associated markers F4/80 and CD11b in PMs from Axl^−/−^ mice compared with WT controls (Figure 1H,I), indicating impaired macrophage maturation. In addition, PMs from Axl‐deficient mice showed a trend toward increased Ly6C expression (*p* = 0.0687) (Figure 1J), suggestive of an enrichment of less mature, monocyte‐like cells within the PF. Consistently, MHCII expression was also significantly reduced in PMs From Axl^−/−^ mice (Figure 1L), pointing to a diminished acquisition of an antigen‐presenting phenotype, whereas CD206 levels remained unaffected (Figure 1K). Collectively, these data demonstrate that global Axl deficiency disrupts the phenotypic composition of the peritoneal macrophage compartment.

**FIGURE 1:**
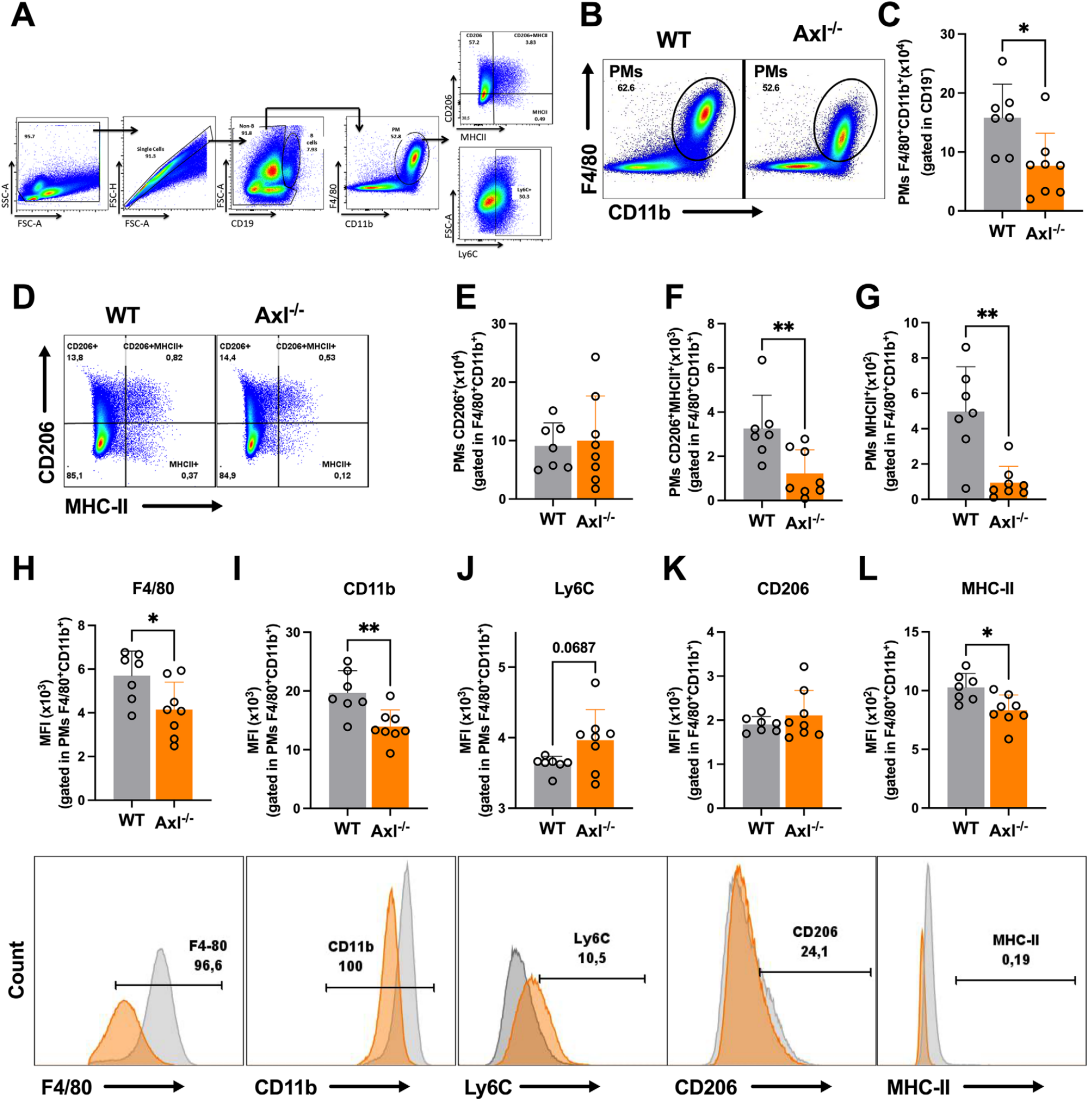
Homeostatic regulation of peritoneal macrophage subsets is dependent on Axl. (A) Gating strategy used to identify peritoneal macrophages (PMs) from peritoneal fluid by flow cytometry. (B) Representative dot plot showing the total PM population identified based on CD11b and F4/80 expression. (C) Quantification of total PMs in wild‐type (WT) and Axl‐deficient (Axl^−/−^) mice. (D) Representative dot plot showing PM subsets based on CD206 and MHCII expression. (E–G) Quantification of PM subsets in WT and Axl^−/−^ mice. (H–L) Mean fluorescence intensity (MFI) of F4/80, CD11b, Ly6C, CD206, and MHC II expression in PMs, along with representative flow cytometry histograms. Data are presented as mean ± SD from one experiment (WT, *n* = 7; Axl^−/−^, *n* = 8). Statistical analysis was performed using Student's *t*‐test; *p* < 0.05.

### 2.2 Axl Deficiency Alters the Metabolic and Oxidative Profile of Peritoneal Macrophages

The phenotypic alterations observed in PMs suggest a shift toward a less mature and functionally reprogrammed PM (Figure 1). Given that macrophage maturation and functional specialization are tightly linked to distinct metabolic programs, proinflammatory‐like cells are predominantly glycolytic, and anti‐inflammatory‐like cells depend on oxidative phosphorylation and the tricarboxylic acid (TCA) cycle [30]. We next examined whether these phenotypic changes were associated with alterations in metabolic and effector functions. PMs from Axl^−/−^ mice exhibited significantly reduced nitrite levels after 24 and 48 h under unstimulated conditions (Figure 2A), indicating impaired basal nitric oxide production. In contrast, lactate accumulation in the culture supernatants of Axl^−/−^ PMs was significantly increased at both time points (Figure 2B), suggesting a shift toward enhanced glycolytic metabolism. This phenotype was further amplified following LPS stimulation, with Axl^−/−^ PMs producing markedly higher levels of lactate compared with WT controls (Figure 2C). Importantly, lactate dehydrogenase (LDH) measurements confirmed that the increased lactate levels were not due to cell death, indicating that these metabolic changes were intrinsic to macrophage nitrite production (Figure 2D).

**FIGURE 2:**
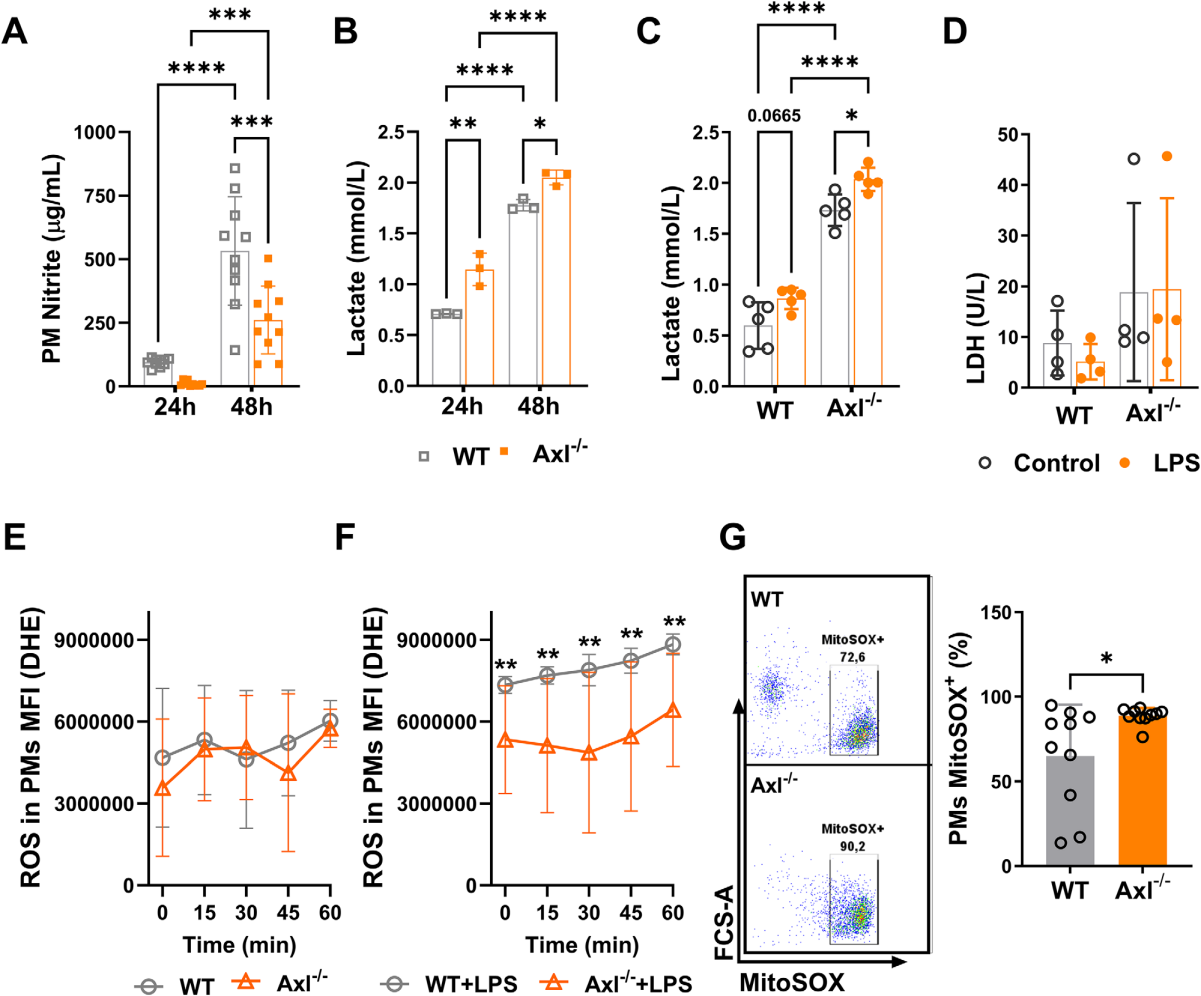
Axl deficiency alters the metabolic and oxidative profile of peritoneal macrophages. (A) Nitrite production by peritoneal macrophages (PMs) from wild‐type (WT) and Axl‐deficient (Axl^−/−^) mice under unstimulated conditions after 24 and 48 h. Data are presented as mean ± SD compiled from three independent experiments. (B) Lactate accumulation in culture supernatants under unstimulated conditions after 24 and 48 h. (C) Lactate levels in PM culture supernatants after 24 h of LPS stimulation. (D) Lactate dehydrogenase (LDH) levels in culture supernatants following 24 h of LPS stimulation, used to assess cell viability. (E) Reactive oxygen species (ROS) production by PMs over 60 min under unstimulated conditions, measured using dihydroethidium (DHE). (F) ROS production by PMs over 60 min following LPS stimulation, measured using DHE. (G) Percentage of MitoSOX‐positive PMs. (H) Mean fluorescence intensity (MFI) of MitoSOX Red within the MitoSOX‐positive PM population, reflecting mitochondrial ROS levels per cell. (B–G) Data are presented as mean ± SD from three independent experiments. Statistical significance was determined using Student's *t*‐test for panels (E–H) and one‐way ANOVA followed by Tukey's multiple comparisons test for panels A–D (*p* < 0.05; *p < 0.01; **p < 0.001; ***p < 0.0001).

Assessment of ROS production revealed no significant differences between WT and Axl^−/−^ PMs under basal conditions (Figure 2E). However, upon LPS stimulation, WT PMs exhibited a robust increase in ROS production over a 60 min period, whereas Axl^−/−^ PMs generated significantly lower levels of ROS (Figure 2F). Analysis of mitochondrial ROS using MitoSOX Red revealed that Axl^−/−^ PMs exhibited an increased frequency of MitoSOX‐positive cells (Figure 2G,H), consistent with elevated mitochondrial ROS levels in Axl‐deficient peritoneal macrophages. Collectively, these data demonstrate that Axl is essential for maintaining the metabolic and functional profile of peritoneal macrophages, linking alterations in macrophage phenotype to impaired effector responses.

### 2.3 Selective Disruption of Glycolytic Intermediates Reveals Impaired Metabolic Adaptability in Axl^−/−^ Macrophages

To further characterize the metabolic reprogramming associated with global Axl deficiency in PMs, we performed an untargeted metabolomic analysis. Unsupervised hierarchical clustering of the metabolomic data revealed a clear separation between WT and Axl^−/−^ PMs, indicating a global remodeling of the metabolic profile in the absence of Axl signaling (Figure 3A). Consistently, principal component analysis (PCA) demonstrated distinct clustering of WT and Axl‐deficient samples along the principal components, confirming that Axl^−/−^ PMs occupy a metabolically distinct state (Figure 3B). Metabolites contributing most strongly to group separation were predominantly associated with glycolysis, energy metabolism, and redox balance, in line with the metabolic and functional alterations observed in previous analyses. Based on these global differences, we next examined the individual variation of key metabolites to identify specific pathways underlying the metabolic reprogramming of Axl^−/−^ PMs.

**FIGURE 3:**
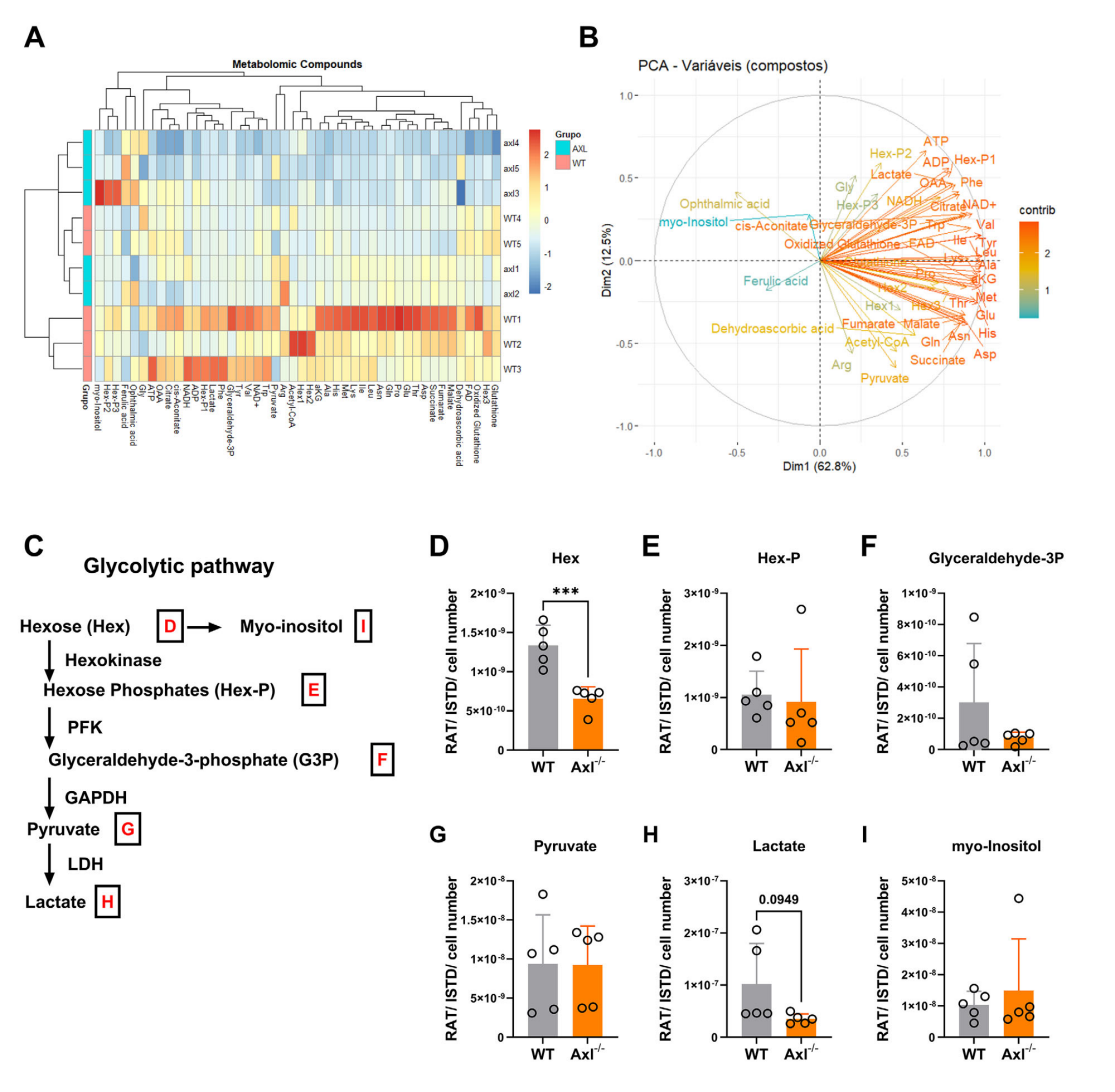
Selective disruption of glycolytic intermediates reveals impaired metabolic adaptability in Axl^−/−^ peritoneal macrophages. (A) Unsupervised hierarchical clustering of metabolomic profiles from PMs isolated from wild‐type (WT) and Axl‐deficient (Axl^−/−^) mice. (B) Principal component analysis (PCA) showing separation between WT and Axl^−/−^ PMs based on metabolic signatures. (C) Schematic representation of glycolytic intermediates analyzed in PMs. (D–I) Quantification of glycolytic intermediates in WT and Axl^−/−^ PMs, including hexose (D), hexose phosphate (Hex‐P) (E), glyceraldehyde‐3‐phosphate (G3P) (F), pyruvate (G), lactate (H), and myo‐inositol (I). Metabolomic results are expressed as ratio area target normalized to the internal standard and cell number (RAT/ISTD/cell number). Data are presented as mean ± SD from one representative experiment (WT and Axl^−/−^, *n* = 5). Statistical analysis was performed using Student's *t*‐test; *p* < 0.05.

To further understand the metabolic dysregulation in Axl^−/−^ PMs, we performed a targeted metabolomic analysis focusing on glycolytic intermediates (Figure 3C). Axl deficiency resulted in a selective reduction in intracellular hexose (Hex) levels (Figure 3D), but not a significant reduction in intracellular lactate abundance (Figure 3H). In contrast, the levels of other glycolytic intermediates, including hexose phosphates (Hex‐P), glyceraldehyde‐3‐phosphate (G3P), pyruvate, and myo‐inositol remained unchanged between WT and Axl^−/−^ PMs (Figure 3E–G,I). These findings indicate that global Axl deficiency does not cause a uniform alteration across the glycolytic pathway but instead selectively affects specific nodes of glucose metabolism.

### 2.4 Axl Regulates Mitochondrial Metabolism in Peritoneal Macrophages

Given the alterations observed in glycolytic intermediates, we next investigated the TCA cycle to determine whether mitochondrial metabolism was also affected in Axl^−/−^ PMs (Figure 4A). Axl^−/−^ macrophages displayed markedly reduced acetyl‐CoA levels (Figure 4B), indicating impaired substrate availability for mitochondrial oxidation or inefficient entry of acetyl groups into the TCA cycle. Consistent with this deficit, early‐cycle intermediates, including citrate and cis‐aconitate, were significantly decreased (Figure 4C,D), demonstrating disruptions at the initial steps of carbon flux through the TCA pathway. These changes were accompanied by pronounced reductions in major redox cofactors: NAD^+^, NADP^+^, NADPH, and FAD (Figure 4K,Q–S), and in ATP (Figure 4N–P), collectively indicating compromised oxidative metabolism, diminished electron‐transfer capacity, and impaired energy production. In addition, α‐ketoglutarate (αKG) was significantly reduced (Figure 4E), further supporting defective mitochondrial carbon processing. In contrast, the abundance of downstream intermediates such as succinate, fumarate, malate, oxaloacetate (OAA), and itaconate remained unchanged (Figure 4F–J), suggesting that later stages of the TCA cycle are relatively preserved despite upstream bottlenecks. Supporting a compensatory metabolic response, several amino acids, including leucine, valine, methionine, alanine, histidine, phenylalanine, tyrosine, and tryptophan, were significantly depleted in Axl^−/−^ PMs (Figure 4U), consistent with increased anaplerotic amino acid catabolism to maintain mitochondrial flux under conditions of impaired acetyl‐CoA availability. Together, these findings reveal that global Axl deficiency induces profound defects in early TCA cycle entry and redox cofactor homeostasis, prompting compensatory amino acid utilization that partially sustains downstream mitochondrial reactions but fails to restore optimal oxidative metabolism.

**FIGURE 4:**
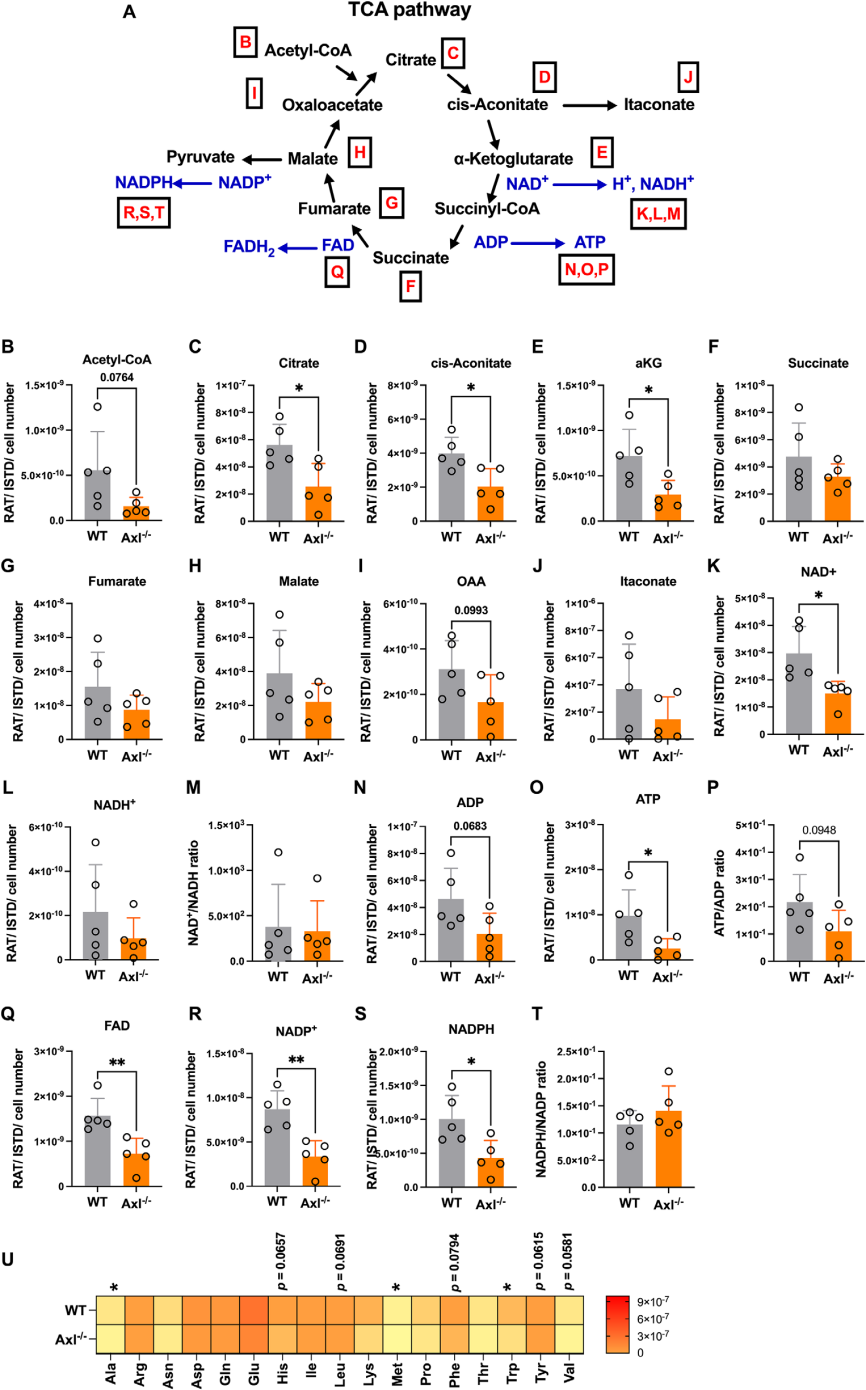
Axl deficiency disrupts TCA cycle entry, redox cofactors, and amino acid metabolism in peritoneal macrophages. (A) Schematic representation of the tricarboxylic acid (TCA) cycle intermediates analyzed in peritoneal macrophages (PMs). Quantification of TCA cycle metabolites in wild‐type (WT) and Axl‐deficient (Axl^−/−^) PMs, including acetyl‐CoA (B), citrate (C), cis‐aconitate (D), α‐ketoglutarate (αKG) (E), succinate (F), fumarate (G), malate (H), oxaloacetate (OAA) (I), and itaconate (J). Quantification of energy‐ and redox‐related metabolites: NAD^+^ (K), NADH (L), NAD^+^/NADH ratio (M), ADP (N), ATP (O), ATP/ADP ratio (P), FAD (Q), NADP^+^ (R), NADPH (S), and NADPH/NADP^+^ ratio (T). (U) Heatmap showing relative abundance of amino acids in WT and Axl^−/−^ PMs. Metabolomic results are expressed as ratio area target normalized to the internal standard and cell number (RAT/ISTD/cell number). Data are presented as mean ± SD from one representative experiment (WT and Axl^−/−^, *n* = 5). Statistical analysis was performed using Student's *t*‐test; *p* < 0.05.

### 2.5 Axl Deficiency Impairs Mitochondrial Maximal Respiratory Capacity in Peritoneal Macrophages

Given that Axl**^−/−^** PMs exhibit increased glycolytic activity and reduced nitric oxide production (Figure 2), and that the broad suppression of early TCA intermediates, redox cofactors, and high‐energy nucleotides (Figure 4) indicates a substantial limitation in mitochondrial oxidative capacity, we next asked whether these biochemical alterations translate into functional mitochondrial impairment. To address this, we evaluated the ability of Axl**^−/−^** PMs to adapt their metabolism when oxidative phosphorylation is challenged. We first assessed glycolytic compensation during impaired electron transport by measuring lactate production following treatment with rotenone, a Complex I inhibitor. In WT PMs, rotenone triggered a rapid and robust increase in lactate release over 60 min, consistent with a compensatory glycolytic shift that maintains ATP generation and regenerates NAD^+^ when NADH oxidation is blocked (Figure 5A). In contrast, Axl**^−/−^** PMs failed to proportionally elevate lactate levels under the same conditions, indicating reduced metabolic flexibility and an impaired ability to engage glycolysis in response to mitochondrial stress. To determine whether Axl deficiency alters mitochondrial respiratory function, oxygen consumption was measured in WT and Axl^−/−^ PMs by high‐resolution respirometry. Representative oxygen flux traces showed a markedly blunted response to FCCP in Axl^−/−^ PMs compared with WT controls (Figure 5B–D). In contrast, maximal respiration was significantly reduced in Axl^−/−^ PMs (Figure 5E), accompanied by a marked decrease in spare respiratory capacity (Figure 5F), indicating impaired mitochondrial reserve capacity and a diminished ability to augment oxidative metabolism in response to increased energetic demand. Notably, proton leak‐associated respiration was significantly increased in Axl^−/−^ PMs (Figure 5G), whereas residual oxygen consumption was unchanged (Figure 5H). Importantly, FCCP‐stimulated respiration reflects not only electron transport system capacity but can also depend on substrate availability and the ability to sustain metabolic flux into the TCA cycle [31]. These findings raised the possibility that the reduced maximal respiration and spare respiratory capacity observed in Axl^−/−^ PMs could reflect alterations in substrate availability in addition to intrinsic changes in mitochondrial respiratory function. To determine whether substrate availability contributed to the impaired respiratory phenotype of Axl^−/−^ PMs, they were subjected to substrate deprivation followed by metabolic rescue with glucose, pyruvate, and GlutaMAX. Substrate deprivation significantly reduced basal respiration in WT PMs, whereas basal respiration was unaffected in Axl^−/−^ PMs (Figure 5I). Repletion with these substrates partially restored basal respiration in both genotypes, resulting in comparable oxygen consumption rates following rescue. Notably, substrate deprivation markedly reduced maximal respiratory capacity in WT PMs (Figure 5J). Notably, the maximal respiratory rates reached by starved WT cells closely resembled those observed in untreated Axl^−/−^ PMs, suggesting that the respiratory phenotype associated with Axl deficiency mimics, at least in part, the metabolic consequences of substrate limitation. Following substrate repletion, maximal respiration increased in both genotypes, although WT PMs still exhibited higher respiratory rates than Axl^−/−^ cells. Consistent with these findings, spare respiratory capacity also tended to decrease following substrate deprivation in WT PMs and was only partially restored upon substrate repletion (Figure 5K). Together, these data suggest that the attenuated FCCP‐stimulated respiration in Axl^−/−^ PMs may not solely reflect an intrinsic limitation in electron transport system capacity, but may also arise from insufficient substrate availability to support oxidative metabolism under conditions of increased energetic demand, as proposed for FCCP‐driven respiratory responses [31]. The convergence of substrate‐deprived WT and Axl^−/−^ PMs on a similar respiratory phenotype further supports a role for Axl in maintaining the metabolic flexibility required to sustain maximal mitochondrial respiration. [31]

**FIGURE 5:**
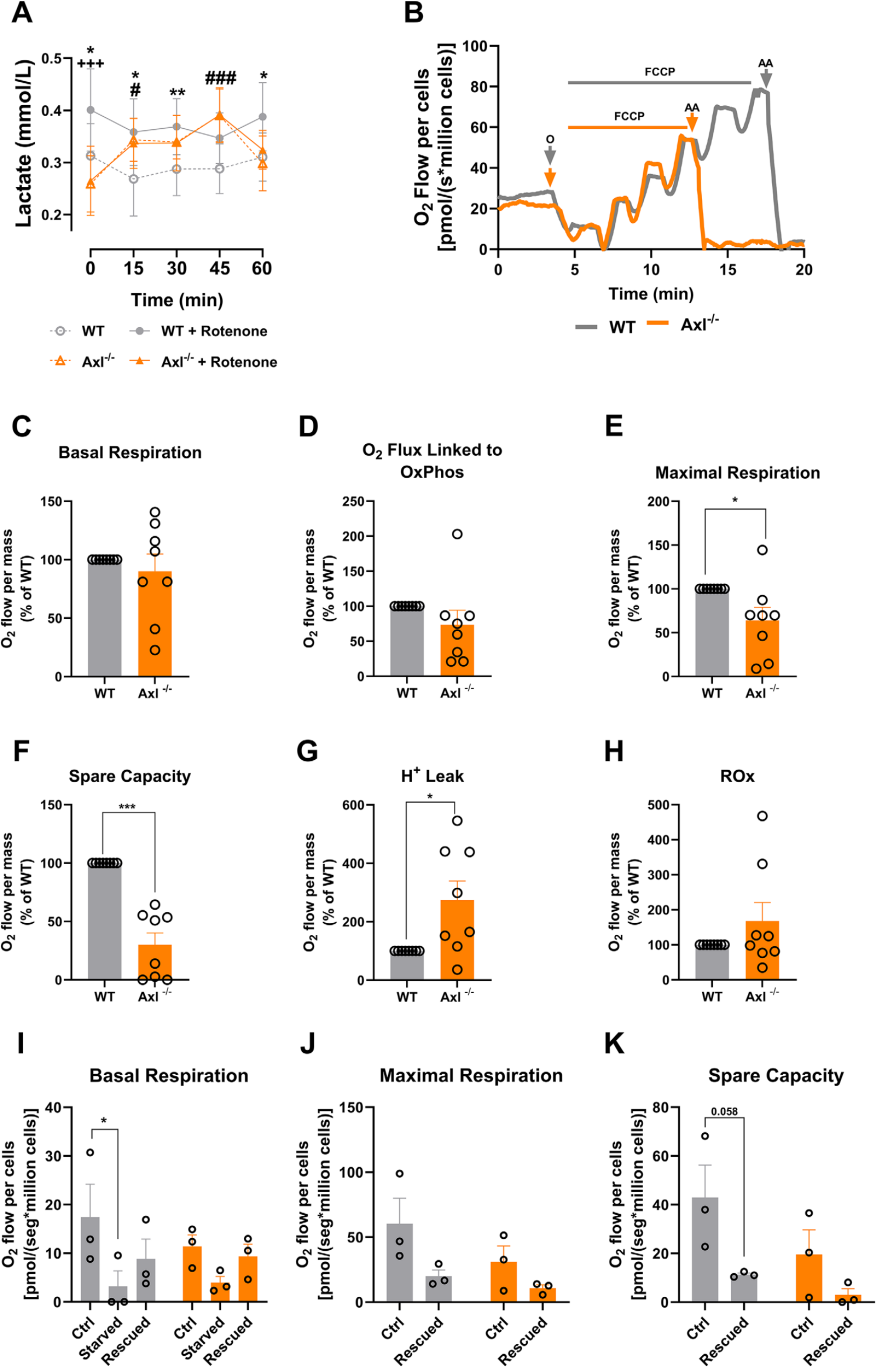
Axl deficiency reduces glycolytic flexibility and impairs maximal mitochondrial respiration and spare respiratory capacity in peritoneal macrophages and phenocopies substrate deprivation. (A) Lactate accumulation in culture supernatants of PMs from wild‐type (WT) and Axl^−/−^ mice following rotenone treatment over a 60 min period. Data are presented as mean ± SD from three independent experiments combined (WT and Axl^−/−^, *n* = 9). Statistical analysis was performed using one‐way ANOVA followed by Tukey's multiple comparisons test (*p < 0.05, **p < 0.01, ***p < 0.001, ****p < 0.0001). *WT vs. WT Rotenone; +WT Rotenone vs Axl^−/−^ Rotenone; #WT vs Axl^−/−^; $Axl^−/−^ vs. Axl^−/−^ Rotenone. (B) Representative high‐resolution respirometry traces of oxygen consumption in WT and Axl^−/−^ peritoneal macrophages. Quantification of (C) basal respiration, (D) OXPHOS‐linked respiration, (E) maximal respiration, (F) spare respiratory capacity, (G) proton leak‐associated respiration, and (H) residual Oxygen consumption (ROx). Quantification os (I) basal respiration, (J) maximal respiration, and (K) spare respiratory capacity in WT and Axl^−/−^ macrophages under control, starved, and rescued conditions. Data are expressed as percentage of WT values. Axl deficiency significantly reduced maximal respiratory capacity, whereas basal respiration, OXPHOS‐linked respiration, and ROx were not significantly altered. Proton leak respiration was increased in Axl^−/−^ macrophages. Quantification of (G) basal respiration, (H) maximal respiration, and (I) spare respiratory capacity in WT and Axl^−/−^ macrophages under control, starved, and rescued conditions. Bars represent mean ± SEM, and individual symbols represent independent biological replicates. Statistical analyses were performed using unpaired Student's *t*‐test (B–G) or two‐way ANOVA followed by Sidak's multiple‐comparison test (H–J). *p < 0.05, ***p < 0.001. Exact *p*‐values are shown when appropriate.

### 2.6 Axl Deficiency Impairs Glutathione Metabolism and Antioxidant Defenses in PMs

Since NADP^+^/NADPH, key cofactors in antioxidant defense [32], were reduced in Axl**^−/−^** PMs (Figure 5R,S), we next assessed metabolites directly involved in redox homeostasis. Reduced glutathione (GSH) was significantly decreased (Figure 6A), whereas the other components showed no significant changes (Figure 6B,C), indicating compromised glutathione metabolism. In contrast, ferulic acid and ophthalmic acid were increased (Figure 6D–E); however, these differences did not reach statistical significance. Nevertheless, these trends may be suggestive of a more oxidatively stressed state in Axl**^−/−^** PMs, consistent with a model in which loss of Axl could contribute to perturbations in central metabolism and redox homeostasis, increasing metabolic vulnerability under stress conditions.

**FIGURE 6:**
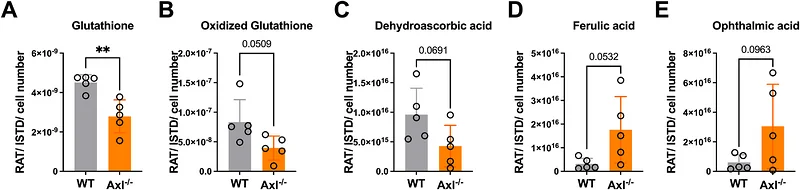
Selective Disruption of Glycolytic Intermediates and Impaired Antioxidant Defense in Axl^−/−^ Macrophages. Levels of antioxidant‐related metabolites in PMs from wild‐type (WT) and Axl‐deficient (Axl^−/−^) mice, including reduced glutathione (GSH) (A), oxidized glutathione (GSSG) (B), dehydroascorbic acid (C), ferulic acid (D), and ophthalmic acid (E). Metabolomic results are expressed as ratio area target normalized to the internal standard and cell number (RAT/ISTD/cell number). Data are presented as mean ± SD from one representative experiment (WT and Axl^−/−^, *n* = 5). Statistical analysis was performed using Student's *t*‐test; *p* < 0.05.

### 2.7 Redox and Metabolic Dysfunction Extend to the Liver in the Absence of Axl Signaling

Given the redox imbalance and impaired antioxidant capacity in Axl^−/−^ PMs, we next investigated whether these alterations extended to other tissues within the peritoneal environment. We focused on the liver, a central organ in metabolic and redox regulation [33], by assessing markers of oxidative stress and antioxidant defenses in hepatic homogenates from WT and Axl^−/−^ mice. Although the levels of lipid peroxidation products, including thiobarbituric acid reactive substances (TBARS) and lipid hydroperoxides, were not reduced in Axl^−/−^ livers (Figure 7A,B), protein carbonylation was significantly increased (Figure 7C). In addition, thiol content and superoxide dismutase (SOD) activity were diminished (Figure 7D,F), while total antioxidant capacity (TAC) and catalase were elevated (Figure 7E,G), consistent with a compensatory response to heightened protein oxidation. Indicators of hepatic dysfunction accompanied these redox changes, including increased glycogen, reduced total protein, and elevated γ‐glutamyl transferase (γ‐GT) activity (Figure 7H–J). Together, these data indicate that Axl signaling is required for systemic redox and metabolic homeostasis. Although its loss drives cell‐intrinsic metabolic dysfunction in PMs, the associated oxidative stress phenotype extends to the liver, highlighting a broader role for Axl in coordinating immune and metabolic responses under homeostatic conditions.

**FIGURE 7:**
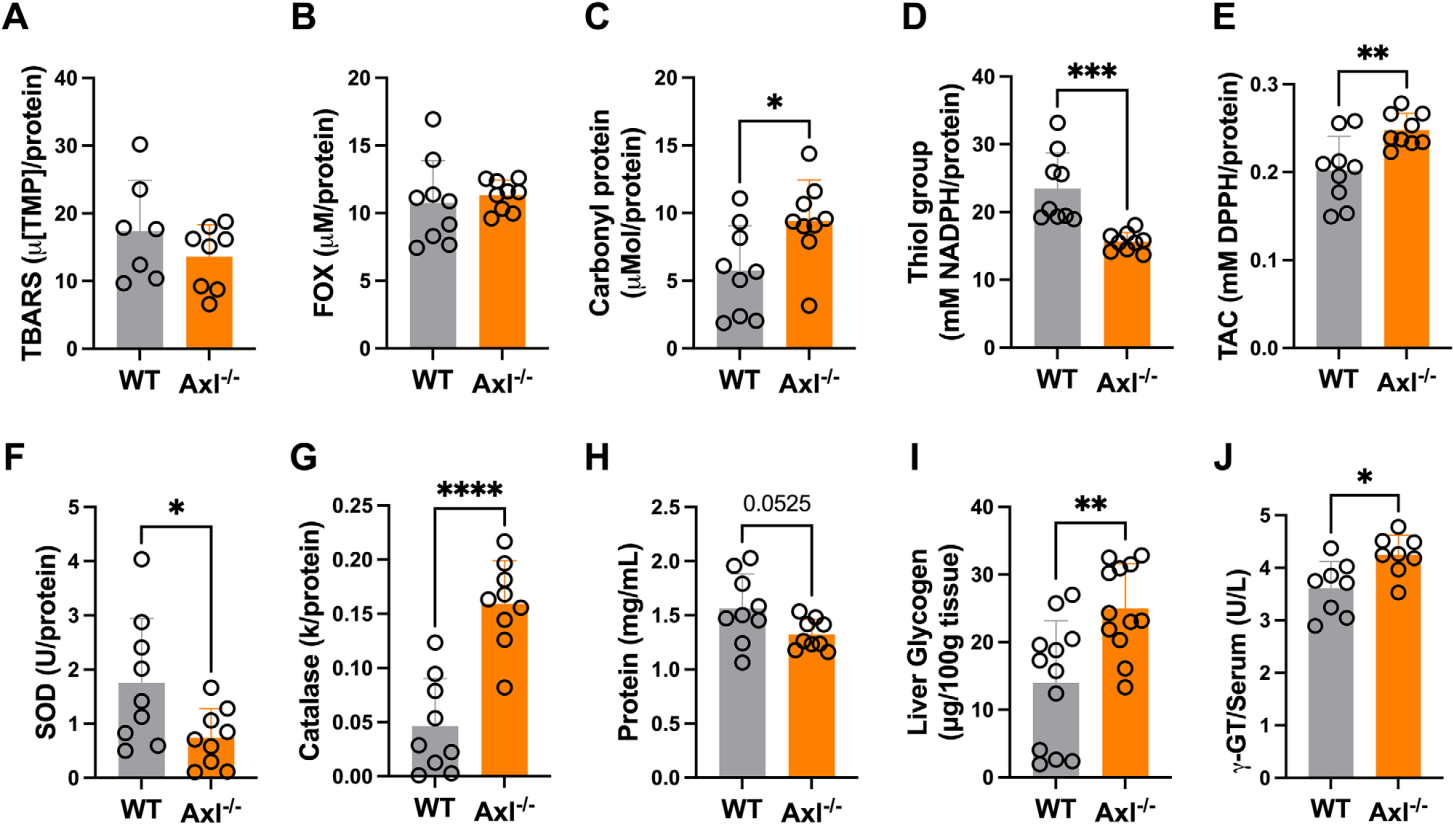
Redox and metabolic dysfunction extend to the liver in the absence of Axl signaling. Quantification of lipid peroxidation markers, including TBARS (A) and lipid hydroperoxides measured by the FOX assay (B), protein carbonylation (C), and hepatic thiol content (D) in liver tissue from wild‐type (WT) and Axl‐deficient (Axl^−/−^) mice. Antioxidant enzyme activities, including superoxide dismutase (SOD) (F) and catalase (G), as well as total antioxidant capacity (TAC) (E), were assessed. Metabolic markers analyzed were total protein content (H), glycogen (I), and γ‐glutamyl transferase (γ‐GT) activity (J). Data are presented as mean ± SD from two independent experiments combined in the graphs (WT and Axl^−/−^, *n* = 8–10). Statistical analysis was performed using Student's *t*‐test; *p* < 0.05.

## 3 Discussion

Macrophages rely on tightly coordinated metabolic and phenotypic programs to maintain tissue homeostasis, integrate inflammatory cues, and execute effector functions across distinct microenvironmental contexts [25, 34]. Among these, the TAM receptor Axl has emerged as a key regulator of efferocytosis, tissue repair, and cell survivor [10, 11, 12, 35, 36]. In this study, we demonstrate that Axl deficiency disrupts multiple, interdependent layers of peritoneal macrophage physiology, encompassing population dynamics, phenotypic maturation, functional responses, and bioenergetic capacity. Axl^−/−^ peritoneal macrophages exhibit altered expression of homeostatic markers, impaired glycolytic and TCA cycle metabolite profiles, and a pronounced defect in maximal mitochondrial respiratory capacity. These cell‐intrinsic perturbations are accompanied by elevated oxidative stress within the peritoneal compartment, marked by increased protein oxidation and diminished antioxidant reserves in the liver, indicating that Axl governs a broader immune‐metabolic axis extending beyond macrophages themselves. Collectively, our findings position Axl as a key coordinator of peritoneal immune equilibrium and systemic redox homeostasis and provide mechanistic insights into how defects in efferocytic signaling propagate metabolic stress across tissues.

Consistent with this overarching disruption of macrophage homeostasis, Axl^−/−^ peritoneal macrophages also display marked alterations in their basal composition and phenotypic identity, characterized by reduced cell numbers and diminished expression of resident macrophage markers such as F4/80 and CD11b, accompanied by increased Ly6C. This shift toward a less mature [1, 37], more monocyte‐like phenotype aligns with their blunted functional responsiveness in vitro. Functionally, Axl^−/−^ PMs produce significantly less nitrite during homeostasis, suggesting impaired acquisition of inflammatory competence or reduced sensitivity to activation signals [38].

These deficits are further reinforced by their diminished ability to generate ROS following LPS stimulation, including both total cellular ROS and mitochondrial ROS detected by MitoSOX, indicating impaired activation of redox‐dependent effector mechanisms [4, 39]. These phenotypic and functional impairments are paralleled by substantial metabolic reprogramming. Axl^−/−^ mice exhibit elevated lactate levels, particularly after LPS challenge, indicating preferential engagement of anaerobic glycolysis and a heightened reliance on a Warburg‐like metabolic profile [40, 41]. Together, these findings further support the notion that Axl governs not only differentiation and inflammatory competence but also the metabolic and redox states of peritoneal macrophages, coordinating their ability to integrate environmental cues into appropriate immune and bioenergetic outputs.

Building on these alterations in inflammatory competence and early bioenergetic responses, the metabolomic analysis further demonstrates that Axl deficiency profoundly reshapes central carbon metabolism in peritoneal macrophages. Despite the reduction of multiple glycolytic intermediates, pyruvate levels remained preserved, a finding suggestive of compensatory replenishment through alanine catabolism, as indicated by the marked decrease in alanine [42]. This pattern indicates that Axl^−/−^ PMs do not experience a complete glycolytic blockade but instead engage alternative substrate routes, such as alanine transamination, to sustain pyruvate availability and lactate output. Such substrate rerouting has been described in metabolically stressed myeloid cells, which maintain pyruvate pools through transaminase‐dependent anaplerosis when glycolytic throughput is insufficient [43]. Consistent with this model, the depletion of key TCA intermediates, including acetyl‐CoA, citrate, cis‐aconitate, and α‐ketoglutarate, indicates a diminished capacity to channel carbon substrates into oxidative metabolism [44]. Because α‐ketoglutarate functions not only as a TCA intermediate but also as a cofactor for dioxygenases controlling HIF‐1α stability and epigenetic remodeling, its reduction may further constrain both redox balance and anti‐inflammatory programming [45], in line with reports showing that αKG deficiency skews macrophages toward a stressed, pro‐oxidant phenotype. Consistent with this interpretation, the depletion of several amino acids in Axl^−^/^−^ PMs suggests increased anaplerotic amino acid catabolism to sustain mitochondrial metabolism under conditions of impaired TCA cycle entry. Alanine may support pyruvate replenishment through transamination reactions, whereas branched‐chain amino acids such as valine and leucine can contribute carbon substrates to succinyl‐CoA and acetyl‐CoA generation, respectively. In parallel, aromatic amino acids including phenylalanine and tyrosine may replenish fumarate pools, while histidine can contribute to glutamate and α‐ketoglutarate production. Together, these findings support the idea that Axl^−^/^−^ PMs engage compensatory amino acid utilization to partially maintain residual TCA cycle flux and mitochondrial function despite reduced oxidative metabolism, a metabolic adaptation commonly observed under conditions of mitochondrial stress and limited carbon availability [46, 47].

These metabolic constraints are functionally reflected in the mitochondrial respirometry profile of Axl^−/−^ PMs. The marked reduction in maximal respiration demonstrates a limited ability to elevate oxidative phosphorylation in response to uncoupling, indicating deficient mitochondrial metabolic flexibility, a phenotype frequently observed in macrophages with impaired TCA flux or defective electron transport chain function [48]. Although respiratory parameters were normalized to WT values for visualization purposes, analysis of the absolute oxygen flux values yielded the same overall conclusions, confirming that the observed reductions in maximal respiration and spare respiratory capacity are not artifacts of data normalization. The concomitant increase in proton leak and the modest reduction in residual (nonmitochondrial) respiration further point to reduced coupling efficiency, suggesting that electrons reaching the respiratory chain are used less effectively to drive ATP synthesis [49]. This integrated deficit is fully compatible with the metabolomic findings: reduced TCA intermediates limit the generation of NADH and FADH_2_, thereby restricting electron supply to the respiratory chain during energetic demand [50]. Notably, substrate deprivation reduced maximal respiration in WT macrophages to levels approaching those observed in untreated Axl^−/−^ cells, supporting the idea that impaired substrate availability contributes to the diminished FCCP‐stimulated respiration observed in Axl‐deficient macrophages. Together with the previously observed elevation in lactate, these data indicate that Axl^−/−^ PMs rely disproportionately on glycolysis while lacking the oxidative reserve needed to adapt their metabolism under stress or activation.

In aggregate, the convergence of metabolomic and functional measurements reveals a dual metabolic constraint imposed by Axl deficiency: attenuated mitochondrial oxidative metabolism coupled with enforced glycolytic dependence. This configuration not only limits the bioenergetic adaptability of peritoneal macrophages but also reinforces their redox vulnerability, providing a mechanistic link to the diminished ROS production observed after LPS stimulation and the systemic oxidative alterations detected in the liver. Overall, these findings position Axl as a central regulator of mitochondrial competence, substrate flexibility, and redox homeostasis in tissue‐resident macrophages.

Complementing the defects in central carbon metabolism and mitochondrial function, Axl deficiency also imposes a profound disruption of redox homeostasis in peritoneal macrophages. The marked reduction in NADP^+^/NADPH and in both reduced and oxidized glutathione (GSH/GSSG) demonstrates a diminished capacity to buffer reactive species and maintain an appropriate intracellular redox tone. The concomitant depletion of dehydroascorbic acid further highlights impaired ascorbate recycling, a critical antioxidant pathway [4]. These deficits not only diminish antioxidant reserves but constrain the activity of NADPH‐dependent enzymes essential for detoxifying ROS [51]. Consistent with this biochemical profile, Axl^−/−^ PMs generate significantly less ROS upon LPS stimulation and exhibit reduced mitochondrial superoxide production as measured by MitoSOX, indicating an inability to mount appropriate oxidative bursts during activation. Paradoxically, metabolites associated with oxidative stress and compensatory responses, such as ophthalmic acid and ferulic acid, were elevated, reinforcing the notion that Axl‐deficient PMs live under chronic redox pressure but lack the antioxidant machinery required to resolve it. When integrated with the impaired TCA flux and reduced respiratory reserve described above, these findings reveal a macrophage population simultaneously energy‐limited and redox‐compromised, unable to efficiently couple metabolic activation to controlled ROS‐mediated antimicrobial and signaling functions.

The metabolic and redox vulnerabilities observed in Axl^−/−^ PMs extend beyond cell‐intrinsic dysfunction and manifest at the systemic level, as evidenced by the oxidative imbalance detected in the liver. Although lipid peroxidation markers were not altered, protein carbonylation was significantly elevated, accompanied by decreased thiol content and reduced SOD activity, hallmarks of persistent oxidative stress and protein damage [52]. The concomitant increase in total antioxidant capacity and catalase suggests a compensatory hepatic response to counteract heightened oxidative burden. Alterations in glycogen content, total protein, and γ‐GT activity further indicate early signs of metabolic and functional dysregulation. Together, these findings underscore that loss of Axl perturbs not only macrophage‐intrinsic pathways but the broader metabolic network of the peritoneal environment, positioning Axl as a critical coordinator of immune, oxidative, and energetic homeostasis across tissues. In this integrated framework, Axl emerges as a central node aligning macrophage maturation, inflammatory competence, metabolic flexibility, and redox balance, ensuring that tissue‐resident macrophages and their surrounding organs maintain functional equilibrium under physiological and stress conditions.

Importantly, these cell‐intrinsic defects propagate systemically, as evidenced by the oxidative and metabolic disturbances detected in the liver, highlighting that Axl functions as a broader coordinator of tissue‐level immune–metabolic homeostasis. Collectively, the integration of phenotypic, metabolomic, and bioenergetic analyses positions Axl as a key determinant of macrophage resilience, enabling these cells to align their metabolic state with environmental cues and inflammatory demands. By delineating the consequences of Axl loss across cellular and tissue scales, this study provides mechanistic insight into how efferocytic receptors safeguard homeostasis and suggests that targeting Axl‐dependent metabolic pathways may offer therapeutic opportunities for conditions characterized by impaired resolution, oxidative imbalance, or macrophage dysfunction.

## 4 Materials and Methods

### 4.1 Mice

Male and female C57BL/6 wild‐type (WT) and Axl^−/−^ mice, aged 8 to 12 weeks, were generously provided by Dr. Brian Kelsall (NIAID, NIH, Bethesda, MD, USA). Animals were maintained in the animal facility of the Universidade Federal do Rio de Janeiro under specific pathogen‐free (SPF) conditions, housed in individually ventilated cages with ad libitum access to food and water, and maintained on a 12 h light/dark cycle. All experimental protocols were reviewed and approved by the Institutional Animal Care and Use Committee (CEUA) of the Universidade Federal do Rio de Janeiro (protocol number 041/17).

### 4.2 Isolation of Peritoneal Macrophages

Resident peritoneal macrophages (PMs) were isolated by peritoneal lavage with 5 mL of cold phosphate‐buffered saline (PBS). The recovered fluid was collected with a syringe, centrifuged (1500 rpm, 15 min, 4 °C), and cells were resuspended in RPMI 1640 medium without phenol red (Gibco, Thermo Fisher Scientific) supplemented with 2 mM L‐glutamine, 50 mM β‐mercaptoethanol (Gibco, Life Techonologies), 100 mM sodium pyruvate (Gibco), 10 µg/mL gentamicin (Sigma‐Aldrich, Cotia, SP, Brazil), amino acids (Gibco), and 5% fetal bovine serum (FBS) (Embriolife). For in vitro assays, cells were plated for 1 h to allow adherence, followed by gentle washing to remove nonadherent cells and debris.

### 4.3 Peritoneal Macrophages Functional Assays and Metabolic Analyses

Adherent macrophages were stimulated to assess nitrite production, lactate, and lactate dehydrogenase (LDH) levels. For nitrites, lactate, and LDH quantification, macrophages were stimulated or not with ultrapure lipopolysaccharide (*E. coli* K12; 2000 ng/mL; Invivogen) for 24 h. Metabolic activity (lactate and LDH production) was further evaluated after treatment with rotenone (1 µg/mL, Sigma) for up to 60 min [53]. For rotenone experiments, macrophages were first allowed to adhere for 1 h, after which the medium was replaced with fresh RPMI containing rotenone. Lactate levels measured immediately after rotenone addition were considered the 0 min time point, and additional supernatants were collected at the indicated time points for subsequent lactate quantification.

### 4.4 MitoSOX Red Mitochondrial Superoxide Detection

Mitochondrial superoxide production was quantified using MitoSOX Red (Thermo Fisher Scientific), a fluorogenic probe that selectively targets mitochondria and fluoresces upon oxidation by superoxide. Peritoneal macrophages were incubated with 5 µM MitoSOX Red in PBS for 10 min at 37°C, protected from light. Cells were then washed twice, resuspended in fresh buffer, and immediately analyzed by flow cytometry. Mean fluorescence intensity (MFI) was used as a measure of mitochondrial ROS levels.

### 4.5 Cytosolic ROS Measurement Using Dihydroethidium (DHE)

Cytosolic reactive oxygen species (ROS) were quantified in PMs using dihydroethidium (DHE; Thermo Fisher Scientific). Cells were seeded in 96‐well plates and incubated with 5 µM DHE diluted in HBSS. Parallel wells were left unstimulated or stimulated with LPS (2000 ng/mL) for 60 min. DHE fluorescence was recorded kinetically using a SpectraMax Paradigm plate reader, with excitation at 520 nm. Five consecutive measurements were acquired at 15 min intervals over the 60 min incubation period. ROS production was expressed as mean fluorescence intensity.

### 4.6 Flow Cytometry

PMs were first incubated with anti‐CD16/CD32 (Fc block, BD Pharmingen, 1:200) for 20 min on ice to block Fc receptors. Cells were then stained for 30 min with the following fluorochrome‐conjugated antibodies: PerCP‐anti‐CD11b (clone M1/70, BioLegend, 1:750), PE‐Cy5‐anti‐CD19 (clone eBio1D3, eBioscience, 1:750), PE‐Cy7‐anti‐F4/80 (clone BM8, BioLegend, 1:250), eFluor450‐anti‐mouse CD206 (clone MR6F36‐10A1, eBioscience, 1:750), and APC‐Cy7‐anti‐I‐A/I‐E (clone M5/114.15.2, BioLegend, 1:750). After staining, cells were washed with PBS containing 2 mM EDTA and 50% FBS. The cells were fixed with 2% paraformaldehyde and acquired on a BD LSRFortessa flow cytometer. Data were analyzed using FlowJo software (Tree Star Inc.).

### 4.7 Nitric Oxide Assay

Nitric oxide production was quantified indirectly by measuring nitrite (NO_2_^−^) accumulation in the cell culture supernatant using the Griess reagent, according to a previously described colorimetric assay [54].

### 4.8 Lactate Measurement

Lactate levels in macrophage culture supernatants were quantified using an enzymatic assay kit (Bioclin, Brazil) according to the manufacturer's instructions. The assay is based on the conversion of L‐lactate to pyruvate by LDH, coupled to NAD^+^ reduction, with absorbance measured at 340 nm. Samples (5 µL) were incubated with 195 µL of working reagent in a 96‐well plate at 37°C for 5 min. Absorbance was read at 340 nm using a Spectra Max Paradigm plate reader (Molecular Devices, USA), and lactate concentration was calculated based on a standard curve.

### 4.9 LDH Activity Assay

LDH activity was determined using a kinetic assay kit (Bioclin, Brazil) by monitoring NADH consumption at 340 nm at 37°C, according to the manufacturer's instructions. Absorbance was recorded over 4 min, and enzyme activity was calculated based on the rate of absorbance decrease (ΔA/min × 8016), expressed in U/L.

### 4.10 Polar Metabolite Extraction and LC‐MS Analysis

Polar metabolites were extracted from 2–8 × 10^6^ PMs using a chloroform:methanol:water (2:2:1.8, v/v/v) solution as previously described [55]. p‐Nitrophenyl phosphate (10 nmol) was added at the beginning of the extraction procedure as an internal standard. Polar fractions were dried under vacuum centrifugation, resuspended in 25 µL of 70% acetonitrile in water, and 10 µL of each sample was injected into a Nexera X2 UHPLC system (Shimadzu) coupled to a Maxis Impact Q‐TOF mass spectrometer (Bruker) equipped with an electrospray ionization (ESI) source. Chromatographic separation was performed on a SeQuant ZIC‐pHILIC column (150 × 2.1 mm, 5 µm particle size; Millipore‐Merck) maintained at 35°C, using a 0.175 mL/min flow rate. A discontinuous gradient of mobile phase A (20 mM ammonium carbonate, pH 9.0 in water) and mobile phase B (acetonitrile) was applied following Ahn et al. (2017): 0–5 min: 95% B; 5–35 min: 95% → 30% B; 35–45 min: 30% B (wash); 45–60 min: 95% B (re‐equilibration). Mass spectra were acquired in negative ion mode, over a 30–1200 m/z range, at 0.67 Hz, without fragmentation. ESI source parameters were set as follows: nebulizer gas (N_2_), 2.0 bar; drying gas (N_2_), 8.0 L/min; source temperature, 200°C; capillary voltage, 4500 V. External mass calibration was performed using a 100 µM sodium formate solution. To support metabolite identification, a pooled quality control sample was analyzed in data‐dependent acquisition (DDA) mode, targeting the top 5 precursor ions per cycle for isolation and fragmentation at 15 and 30 eV collision energies, with all other MS parameters identical to full‐scan acquisitions.

Raw LC–MS data were processed using MZmine 2 [56], and multivariate statistical analyses were performed on the MetaboAnalyst online platform [57, 58]. For targeted analysis of specific metabolites, raw data files were processed in QuantAnalysis 5.0 (Bruker), and univariate statistical analyses were conducted using GraphPad Prism 8.0. Metabolite features were annotated based on accurate masses (10 ppm tolerance) and fragmentation spectra, using the HMDB database [59], mzCloud (www.mzcloud.org), and Metlin [60]. Quantitative results were expressed as the ratio of the metabolite peak area target (RAT) to the internal standard (ISTD), normalized to the total number of cells in each sample (RAT/ISTD/cell number). This normalization strategy was used to minimize technical variability and account for differences in cell input between samples.

### 4.11 High‐Resolution Respirometry Assay

The respirometry experiments were conducted using the Oroboros Oxygraph‐2k system, following protocols previously described [61, 62]. WT and Axl^−/−^ peritoneal macrophages were cultured in RPMI 1640 medium without phenol red and seeded at a density of 1 million cells per chamber in the Oroboros O2k respirometer. The following inhibitors and uncoupler were used as described: 0.5 µg/mL of oligomycin was added to the chamber to inhibit ATP synthase, followed by the uncoupling agent, carbonyl cyanide‐4‐(trifluoromethoxy) phenylhydrazone (FCCP), which was titrated up to a final concentration of 2 µM. Lastly, 4 µM of antimycin A, the inhibitor of mitochondrial complex III, was added to the chamber. The mitochondrial physiology parameters were assessed under specific experimental conditions. Residual oxygen consumption (Rox) was determined after the addition of Antimycin A to establish a baseline for respiratory activity not associated with oxidative phosphorylation (OXPHOS). Basal cellular respiration was computed by subtracting non‐mitochondrial respiration from the basal respiration recorded at the beginning of the experiment, before any of the additions. O_2_ flux linked to OXPHOS was calculated as the difference between basal respiration and oligomycin‐insensitive respiration after correction for residual oxygen consumption. Maximal respiration was determined in the presence of FCCP, with non‐OXPHOS respiration subtracted to isolate the mitochondrial contribution to this maximal activity. Spare respiratory capacity was calculated as the difference between maximal and basal respiration after correction for residual oxygen consumption. Lastly, the measurement of H^+^ leak was performed by evaluating the effects induced by oligomycin on respiration, with the nonmitochondrial component subtracted for precise evaluation. Data were analyzed using Oroboros DatLab software.

### 4.12 Substrate Deprivation and Respiratory Rescue Assay

To evaluate whether limited substrate availability could contribute to the respiratory phenotype observed in Axl‐deficient macrophages, WT and Axl^−/−^ peritoneal macrophages were subjected to acute nutrient deprivation before high‐resolution respirometry analysis. Cells were incubated for 1 h in Dulbecco's modified Eagle's medium (Sigma‐Aldrich). Following nutrient deprivation, macrophages were transferred to the Oroboros O2k respirometer at a density of 1 × 10^6^ cells per chamber. Basal oxygen consumption was initially recorded under substrate‐deprived conditions. Subsequently, 1.6 mM of GlutaMAX supplement (Thermo Fisher Scientific), 25 mM of glucose, and 1 mM sodium pyruvate were added directly to the chamber at the concentrations used in the culture medium. Oxygen consumption was monitored to assess respiratory recovery following substrate replenishment. After stabilization of oxygen flux, mitochondrial respiratory parameters were determined as previously described.

### 4.13 Protein Quantification

Protein levels in tissue homogenates were measured by the Bradford (Thermo Fisher Scientific) assay. Absorbance was read at 595 nm (SpectraMax Paradigm), and concentrations were calculated from the bovine serum albumin (Sigma‐Aldrich) standard curve. Results were used to normalize oxidative stress assays.

### 4.14 Liver Preparation

Liver tissue was collected at euthanasia, aliquoted, and stored at –80°C until the day of analysis. For this, 200 mg of the tissue was homogenized in 1 mL antioxidant buffer (Tris‐HCl 10 mM, pH 8.0, saline 0.9%, aprotinin, PMSF, MilliQ water) using Ultra‐Turrax (T25, IKA, Staufen, Alemanha), kept on ice. Samples were centrifuged (1000 rpm, 15 min, 4°C), supernatants collected and stored at –80°C for oxidative stress and antioxidant enzyme assays [63].

### 4.15 Lipid Peroxidation Assay

Tissue homogenates were mixed with phosphate buffer and 10% tricarboxylic acid, cooled, and centrifuged. Supernatants reacted with thiobarbituric acid substrates (TBAS) and were heated at 100°C for 20 min. After cooling, absorbance was measured at 532 nm (Spectra Max Paradigm). Concentrations were calculated using a TMP (tetramethyl‐p‐phenylenediamine) standard curve and expressed as μ[TMP]/mg protein [64].

### 4.16 Total Hydroperoxide Measurement

Tissue homogenates were mixed with 40 mM butylated hydroxytoluene (BHT) in methanol, vortexed, and centrifuged (6000 rpm, 5 min, 4 °C). Supernatants (20 µL) were incubated with 360 µL of FOX reagent composed of 100 µM xylenol orange, 250 µM ammonium ferrous sulfate, 25 mM sulfuric acid, and 90% ethanol (v/v). After 2 h of incubation in the dark at room temperature, absorbance was measured at 540 and 560 nm using a Spectra Max Paradigm plate reader. Hydroperoxide levels were determined from the absorbance difference (ΔA) and expressed as µM/mg protein [65].

### 4.17 Protein Carbonyl Content Assay

Tissue homogenates were mixed with 2,4‐dinitrophenylhydrazine (DNPH) or phosphate buffer (blank), incubated for 10 min at room temperature in the dark, followed by addition of NaOH and further 10 min incubation. Absorbance was measured at 450 nm (SpectraMax Paradigm). Carbonyl concentration was calculated using the molar extinction coefficient 22,308 M^−^^1^cm^−^^1^ and expressed as µmol/mg protein [66].

### 4.18 Thiol Group Assay

Tissue homogenates were prepared as blanks (sample + thiol buffer + methanol) and test samples (sample + thiol buffer + methanol + DTNB). After 15 min incubation at room temperature and centrifugation (5000 rpm, 15 min, 25°C), absorbance of the NTB_2_^−^ ion was measured at 412 nm (Spectra Max Paradigm). Concentrations were calculated using the molar extinction coefficient 13,600 M^−^^1^cm^−^^1^ and expressed as mM NADPH/mg protein [67].

### 4.19 Total Antioxidant Capacity Assay

Samples (0.5 mL) were mixed with 3 mL absolute ethanol and 0.3 mL of 0.5 mM DPPH (2,2‐difenil‐1‐picrilhidrazila) solution in ethanol. After 100 min incubation, absorbance was measured at 517 nm (Spectra Max Paradigm). Antioxidant activity was calculated from a DPPH standard curve and expressed as mM DPPH [68].

### 4.20 Catalase Activity Assay

Liver homogenates (10 µL) were added to 990 µL phosphate buffer and incubated at 37°C. The reaction was started by adding 5 µL of 30% hydrogen peroxide, and absorbance at 240 nm was measured every 15 s for 90 s (Spectra Max Paradigm). Catalase activity was calculated from the linear decrease in absorbance and expressed as k·mg^−^^1^ protein [69].

### 4.21 Superoxide Dismutase Activity Assay

Enzyme activity was measured by the inhibition of adrenaline auto‐oxidation to adrenochrome at 480 nm. Samples (10 µL) were mixed with glycine buffer (50 mM, pH 10.2) and catalase (0.0024 g/mL), incubated at 32°C for 1 min, then adrenaline (60 mM) was added to start the reaction. Absorbance was recorded every 10 s for 180 s (SpectraMax Paradigm). Activity was calculated by subtracting blank readings (auto‐oxidation without sample) and expressed as U/mg protein [70].

### 4.22 Hepatic Glycogen Quantification

Liver homogenates were assayed for glycogen using a Bioclin enzymatic kit, following manufacturer instructions. Absorbance was measured in a microplate reader and glycogen content calculated from the standard curve.

### 4.23 γ‐Glutamyl Transferase Activity Assay

γ‐glutamyl transferase (γ‐GT) activity was measured in tissue homogenates using a commercial enzymatic kit (Bioclin), following the manufacturer's protocol. Samples were incubated with reagents that generate a colorimetric reaction proportional to γ‐GT activity. Absorbance was read using a microplate reader, and enzyme activity was calculated according to the standard curve.

### 4.24 Statistical Analyses

All statistical analyses were performed using GraphPad Prism software (version 9.0). Comparisons between two groups were conducted using Student's *t*‐test. For analyses involving more than two groups, one‐way analysis of variance (ANOVA) followed by Tukey's multiple comparisons post hoc test was applied. Detailed information regarding statistical significance is provided within the figures and corresponding legends.

## Author Contributions

Conceptualization: J.R.M.F., A.A.F. Investigation: J.R.M.F., T.S.R., M.S.L., K.G.P., L.R.R., I.A.O., V.S.P., A.G., C.M., J.M., A.A.F. Data curation—formal analysis: J.R.M.F., T.S.R., K.G.P., A.G., A.A.F. Project Administration/oversight: A.A.F. Writing–original draft: J.R.M.F., A.A.F. Writing–review, editing, and revision: J.R.M.F., A.L.A.C., V.S.P., J.A.L., A.T., A.G., A.A.F.

## Funding

The Article Processing Charge for the publication of this research was funded by the Coordenação de Aperfeiçoamento de Pessoal de Nível Superior — Brasil (CAPES) (ROR identifier: 00x0ma614).

## Ethics Approval Statement for Human and/or Animal Studies

All experimental protocols were reviewed and approved by the Institutional Animal Care and Use Committee (CEUA) of the Universidade Federal do Rio de Janeiro (protocol number 041/17).

## Conflicts of Interest

The authors declare no conflicts of interest.

## Data Availability

All data supporting the findings of this study are available from the corresponding author upon reasonable request.
